# Effects of a Public Education Campaign on the Association Between Knowledge of Early Stroke Symptoms and Intention to Call an Ambulance at Stroke Onset: The Acquisition of Stroke Knowledge (ASK) Study

**DOI:** 10.2188/jea.JE20150040

**Published:** 2016-03-05

**Authors:** Tomofumi Nishikawa, Tomonori Okamura, Hirofumi Nakayama, Naomi Miyamatsu, Akiko Morimoto, Kazunori Toyoda, Kazuo Suzuki, Akihiro Toyota, Takashi Hata, Takenori Yamaguchi

**Affiliations:** 1 Department of Health Science, Kyoto Koka Women’s University, Kyoto, Japan; 2 Department of Preventive Medicine and Public Health, Keio University, Tokyo, Japan; 3 Japan Stroke Association, Osaka, Japan; 4 Department of Clinical Nursing, Shiga University of Medical Science, Otsu, Japan; 5 Department of Cerebrovascular Medicine, National Cerebral and Cardiovascular Center, Suita, Osaka, Japan; 6 Department of Epidemiology, Research Institute for Brain and Blood Vessels-Akita, Akita, Japan; 7 Rehabilitation Center, Chugoku Rosai Hospital, Kure, Hiroshima, Japan; 8 Department of Neurology, Shizuoka City Shimizu Hospital, Shimizu, Shizuoka, Japan

**Keywords:** ambulance call, knowledge, public education, early stroke symptoms

## Abstract

**Background:**

An immediate ambulance call offers the greatest opportunity for acute stroke therapy. Effectively using ambulance services requires strengthening the association between knowledge of early stroke symptoms and intention to call an ambulance at stroke onset, and encouraging the public to use ambulance services.

**Methods:**

The present study utilized data from the Acquisition of Stroke Knowledge (ASK) study, which administered multiple-choice, mail-in surveys regarding awareness of early stroke symptoms and response to a stroke attack before and after a 2-year stroke education campaign in two areas subject to intensive and moderate intervention, as well as in a control area, in Japan. In these three areas, 3833 individuals (1680, 1088 and 1065 participants in intensive intervention, moderate intervention, and control areas, respectively), aged 40 to 74 years, who responded appropriately to each survey were included in the present study.

**Results:**

After the intervention, the number of correctly identified symptoms significantly associated with intention to call an ambulance (*P* < 0.05) increased (eg, from 4 to 5 correctly identified symptoms), without increasing choice of decoy symptoms in the intensive intervention area. Meanwhile, in other areas, rate of identification of not only correct symptoms but also decoy symptoms associated with intention to call an ambulance increased. Furthermore, the association between improvement in the knowledge of stroke symptoms and intention to call an ambulance was observed only in the intensive intervention area (*P* = 0.009).

**Conclusions:**

Our results indicate that intensive interventions are useful for strengthening the association between correct knowledge of early stroke symptoms and intention to call an ambulance, without strengthening the association between incorrect knowledge and intention to call an ambulance.

## INTRODUCTION

Stroke is one of the leading causes of death and disability worldwide.^1^^–^^3^ Decreasing the time from stroke onset to hospital arrival offers the greatest opportunity for effective acute stroke therapy^4^^–^^8^; however, delay in hospital presentation of patients with acute stroke still remains substantial.^9^^–^^12^

One of the most influential factors for early hospital presentation after recognition of early stroke symptoms is an immediate ambulance call. As a matter of course, urging the public to call an ambulance when someone is suspected of stroke seems to be a proper action to take. However, encouraging the public to utilize ambulances without strengthening the association between correct knowledge of early stroke symptoms and a need to call an ambulance may lead to overuse of ambulances, which can be harmful to the ambulance dispatch system.^17^

The aim of the present study was to analyze the effects of a 2-year educational campaign to improve the knowledge of early stroke symptoms among community residents in the Acquisition of Stroke Knowledge (ASK) study on the association between correct or incorrect knowledge of early stroke symptoms and intention to call an ambulance.^18^

## METHODS

### ASK study

The ASK study was a nonrandomized, community intervention trial that aimed to evaluate the effects of public education about early stroke symptoms and the appropriate response to stroke onset in three Japanese cities: Akita, Shizuoka, and Kure.^18^^,^^19^ Akita City was selected as an intensive intervention area, Kure City was selected as a moderate intervention area, and Shizuoka City was selected as the control area. A 3-month pre-intervention survey (April 2006 to June 2006) was followed by 22 months of community intervention (July 2006 to April 2008). After the community intervention, a 2-month post-intervention survey (May 2008 to June 2008) was performed. In both pre- and post-intervention surveys, a self-administered questionnaire was mailed to each participant. The questionnaire included a question on early stroke symptoms in which the participants were required to identify the correct early stroke symptoms among 10 multiple-choice items, which consisted of 5 correct symptoms and 5 decoys. This study was approved by the ethics committee of Shiga University of Medical Science (17-97).

### Participants

From the three areas, 11 306 (3776 in the intensive intervention area, 3695 in the moderate intervention area, and 3835 in the control area) community residents, aged 40 to 74 years, were randomly selected by an age-stratified random sampling method from the Basic Resident Register, and 5540 individuals responded to the pre-intervention mail-in survey.^19^ Of 5509 individuals who agreed to participate in the post-intervention mail-in survey, 3926 ultimately responded. The response rates were 71.3% overall, 73.8% (1719/2329) in the intensive intervention area, 71.4% (1116/1562) in the moderate intervention area, and 67.4% (1091/1618) in the control area. In the present study, 915 individuals selected all five correct symptoms. Of the 3926 individuals who responded to the post-intervention survey, 71 who did not complete the questionnaire by themselves or did not fill in the required data and 22 who chose all 10 items (including the 5 decoys) as early symptoms of stroke either in the pre- or post-intervention survey were excluded, in accordance with the same exclusion criteria used in a previous report.^18^ Ultimately, 3833 individuals (1680 in the intensive intervention area, 1088 in the moderate intervention area, and 1065 in the control area) were eligible for the present analysis.

### Community intervention

The community intervention was conducted by distribution of leaflets and booklets and by holding lectures.^18^ The leaflets and booklets mentioned early stroke symptoms and the need for appropriate response to stroke onset every time a stroke is suspected. Leaflets and booklets were distributed to all homes in the intensive and moderate intervention areas for 2 years between pre- and post-intervention surveys. In the intensive intervention area, leaflets were distributed 12 times, booklets were distributed twice, and lectures (about the early symptoms and appropriate response to onset of stroke, as well as the risk factors for stroke) were presented 13 times. In the moderate intervention area, leaflets and booklets were distributed once each, and lectures were presented five times. The contents and distribution schedule of the leaflets and booklets are described in the previous report.^18^ The control area did not receive any of these interventions.

### Questionnaire

In both pre- and post-intervention surveys, a self-administered questionnaire was mailed to each participant. The questionnaire consisted of the following: “general knowledge of stroke”, “early symptoms of stroke”, “the response to a stroke attack”, “information sources for knowledge about stroke”, and “sociodemographic factors”.^19^

The details of the question on “early symptoms of stroke” were presented in the previous reports^18^^,^^19^; briefly, there were five correct answers (sudden confusion or trouble speaking or understanding speech; sudden one-sided numbness or weakness of the face, arms, or legs; sudden severe headache with no known cause; sudden trouble with walking, dizziness, or loss of balance or coordination; and sudden visual disturbances in one or both eyes)^20^ and five decoy answers (sudden nasal bleeding, sudden increase in body temperature, sudden pain in the left shoulder, numbness of bilateral fingers, and sudden difficulty in breathing) presented as multiple-choice items. Participants were asked to choose all early stroke symptoms from these 10 multiple-choice items. In the present study, the respondents who were “aware of early stroke symptoms” were defined as those who selected all 5 correct early stroke symptoms from the 10 multiple-choice items, except for those who selected all items.

The question regarding “the response to a stroke attack” consisted of eight multiple-choice items, for which a single answer was required (immediately call an ambulance, immediately call a primary physician at clinic or hospital, immediately call a large and/or special hospital, immediately see a primary physician at a clinic or hospital, immediately see a doctor in a large and/or special hospital, see a primary physician at a clinic or hospital during office hours, see a doctor in a large and/or special hospital during office hours, and wait and observe symptoms for several days). “Intention to call an ambulance” was defined as selecting “immediately call an ambulance” as an answer to the question about what action to take when a stroke is suspected.

A nationwide stroke campaign, with newspaper advertisements about the early stroke symptoms and calling an ambulance as early as possible, was conducted by Advertising Council (AC) Japan during the intervention period, which followed the introduction of thrombolytic therapy with tissue-type plasminogen activator for cerebral infarction. In addition to the above, participants were asked if they had seen the advertisements by AC Japan.

### Statistical analysis

Differences in demographic characteristics and knowledge of early stroke symptoms among the three areas were determined using analysis of variance for age and Pearson’s chi-square test or Fisher’s exact test for dichotomous and categorical data, respectively. Those who responded to the question on “early symptoms of stroke” in both pre- and post-intervention questionnaires were classified into two groups: those who chose all five correct answers (aware) and the rest of the participants (unaware). Those who chose “immediately call an ambulance” as an answer to the question about what action to take when a stroke is suspected were classified into the “would call” group, while those who chose other items were grouped into “other” group. McNemar’s test was used to assess the effect of the 2-year educational campaign on being aware of early stroke symptoms and intention to call an ambulance. The association between each symptom chosen for early stroke symptoms and intention to call an ambulance was evaluated using Fisher’s exact test. Furthermore, the association between improvement in knowledge of early stroke symptoms and intention to call an ambulance after public education in participants who had chosen ≤4 correct symptoms in the pre-intervention survey was also evaluated using Fisher’s exact test. All significance tests were two-tailed, and *P* < 0.05 was considered significant in all analyses. Data were analyzed with SPSS version 19.0 for Windows (IBM Corp., Armonk, NY, USA).

## RESULTS

### Demographics

Demographics in the pre-intervention survey are shown in Table 1. Education level (*P* < 0.001), rate of living alone (*P* = 0.001), and rate of having close contact with stroke patients (*P* < 0.001) differed significantly among participants in the three areas. There were no significant differences in age, sex, history of stroke, and history of transient ischemic attack among participants in the three areas. Statistically significant differences were observed in the knowledge of “sudden confusion or trouble speaking or understanding speech” and “sudden visual disturbances in one or both eyes” among participants in the three areas. The numbers of participants who reported that they had participated in lectures on stroke were 62 (3.6%) in the intensive intervention area, 35 (3.2%) in the moderate intervention area, and 17 (1.5%) in the control area. Since we thought it was difficult for the participants to distinguish between lectures on stroke hosted by us and those hosted by others at the time of the post-intervention survey, we counted all lectures on stroke for which they reported participation.

**Table 1.** Demographics, knowledge of early stroke symptoms and intention to call an ambulance at stroke in the pre-intervention survey

	Akita (Intensive) (*n* = 1680)	Kure (Moderate) (*n* = 1088)	Shizuoka (Control) (*n* = 1065)	Total (*n* = 3833)	*P* value
Age, years^a^	58.3 (9.8)	59.3 (9.6)	58.8 (9.4)	58.7 (9.6)	0.105
Male	751 (44.7)	503 (46.2)	481 (45.2)	1735 (45.3)	0.730
Education, >12 years	248 (14.8)	394 (36.2)	331 (31.1)	973 (25.4)	<0.001
Living alone	105 (6.3)	107 (9.8)	69 (6.5)	281 (7.3)	0.001
History of stroke	42 (2.5)	25 (2.3)	24 (2.3)	91 (2.4)	0.901
History of transient ischemic attack	10 (0.6)	8 (0.7)	7 (0.7)	25 (0.7)	0.905
Presence of patients with stroke living close to the participants	1087 (64.7)	492 (45.2)	559 (52.5)	2138 (55.8)	<0.001
Correct answer about stroke symptoms					
Sudden confusion or trouble speaking or understanding speech	1433 (85.3)	966 (88.8)	949 (89.1)	3348 (87.3)	0.003
Sudden one-sided numbness or weakness of the face, arms, or legs	1490 (88.7)	932 (85.7)	926 (86.9)	3348 (87.3)	0.058
Sudden severe headache with no known cause	1237 (73.6)	794 (73.0)	791 (74.3)	2822 (73.6)	0.793
Sudden trouble with walking, dizziness, or loss of balance or coordination	1112 (66.2)	684 (62.9)	660 (62.0)	2456 (64.1)	0.050
Sudden visual disturbances in one or both eyes	549 (32.7)	411 (37.8)	395 (37.1)	1355 (35.4)	0.009
Aware of early stroke symptoms	378 (22.5)	272 (25.0)	256 (24.0)	906 (23.6)	0.299
Intention to call an ambulance	1405 (83.6)	858 (78.9)	870 (81.7)	3133 (81.7)	0.007

^a^Age was analyzed using ANOVA, and is shown in the mean (standard deviation). Dichotomous and categorical data were analyzed using the Pearson’s chi-square test, and are shown as number (%).

### Knowledge of early stroke symptoms in the pre- and post-intervention surveys

**Figure 1.**
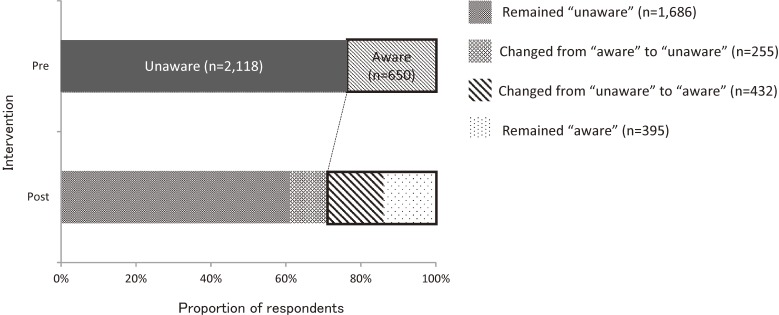
Proportion of respondents who selected five correct answers in the intervention areas (Akita & Kure) in the pre- and the post-intervention surveys.

The change in knowledge of early stroke symptoms by individuals between the pre- and the post-intervention surveys is shown in Table 2. McNemar’s test showed that the knowledge of early stroke symptoms improved significantly in the intensive intervention area (*P* < 0.001) and moderate intervention area (*P* = 0.011), but not in the control area (*P* = 0.088). In the pre-intervention survey, 650 (23.5%) respondents selected all 5 correct answers in the intervention areas. In the post-intervention survey among respondents in the intervention areas, 395 (14.3%) remained in the “aware” category, 255 (9.2%) slipped from “aware” to “unaware”, 1686 (60.9%) remained in the “unaware” category, and 432 (15.6%) improved from “unaware” to “aware” (Figure 1).

**Table 2.** The improvement of the knowledge of stroke symptoms; those who chose 5 correct stroke symptoms and the rest

			Post-intervention			

			unaware	aware	Total	*P* value
Akita (Intensive)	Pre-intervention	unaware	1022 (60.8)	280 (16.7)	1302 (77.5)	<0.001
		aware	145 (8.7)	233 (13.8)	378 (22.5)	
			1167 (69.5)	513 (30.5)	1680	
Kure (Moderate)		unaware	664 (61.0)	152 (14.0)	816 (75.0)	0.011
		aware	110 (10.1)	162 (14.9)	272 (25.0)	
			774 (71.1)	314 (28.9)	1088	
Shizuoka (Control)		unaware	660 (62.0)	149 (14.0)	809 (76.0)	0.088
		aware	120 (11.2)	136 (12.8)	256 (24.0)	
			780 (73.2)	285 (26.8)	1065	

The data were analyzed using the McNemar’s test, and are shown as number (%). aware; those who chose five correct stroke symptoms, unaware; the rest.

### Intention to call an ambulance for early stroke symptoms in the pre- and post-intervention surveys

In the pre-intervention surveys, intention to call an ambulance at stroke onset was reported by 83.6% (1405/1680) of participants in the intensive intervention area, 78.9% (858/1088) of participants in the moderate intervention area, 81.7% (870/1065) of participants in the control area, and 81.7% (3133/3833) overall; in the post-intervention surveys, intention to call an ambulance at stroke onset was reported by 82.0% (1378/1680), 77.5% (843/1088), 79.8% (850/1065), and 80.1% (3071/3833), respectively (Table 3). The proportions of respondents reporting an intention to call an ambulance at stroke onset were significantly different among the three areas both in pre- and post-intervention surveys. There were no significant differences in exposure to the AC Japan campaign between the “would call” group and “other” group in any area; the advertisement by AC Japan had been seen by 46.4% (606/1316) of participants in the “would call” group and 46.0% (127/276) of participants in “other” group in the intensive intervention area (*P* = 0.523), 44.7% (367/821) and 43.4% (102/235) in the moderate intervention area (*P* = 0.391), and 36.9% (307/830) and 42.3% (88/208) in the control area (*P* = 0.092), respectively.

**Table 3.** Change in the proportion of the respondents who would call an ambulance at stroke onset

			Post-intervention			

			Would call	Would not call	Total	*P* value
Akita (Intensive)	Pre-intervention	Would call	1206 (71.8)	199 (11.8)	1405 (83.6)	0.177
		Would not call	172 (10.2)	103 (6.2)	275 (16.4)	
		Total	1378 (82.0)	302 (18.0)	1680	
Kure (Moderate)		Would call	703 (64.6)	155 (14.2)	858 (78.9)	0.415
		Would not call	140 (12.8)	90 (8.3)	230 (21.1)	
		Total	843 (77.5)	245 (22.5)	1088	
Shizuoka (Control)		Would call	739 (69.4)	131 (12.3)	870 (81.7)	0.222
		Would not call	111 (10.4)	84 (7.9)	195 (18.3)	
		Total	850 (79.8)	215 (20.2)	1065	

The data were analyzed using the McNemar’s test, and are shown as number (%). Would call; those who chose “call an ambulance” at the stroke onset, would not call; those who did not choose it.

**Figure 2.**
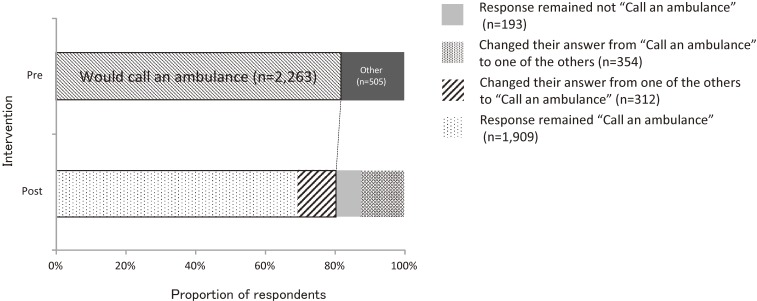
Proportion of respondents who selected “immediately call an ambulance” at stroke onset in the intervention areas (Akita & Kure) in the pre- and the post-intervention surveys.

Comparison of individuals between the pre- and the post-intervention surveys using McNemar’s test revealed no significant change in the proportion of those with an intention to call an ambulance at stroke onset in all three areas (Table 3). In the pre-intervention survey, 81.8% (2263/2767) respondents reported an intention to call an ambulance at stroke onset in the intervention areas. In the post-intervention survey, 69.0% (1909/2767) of respondents in the intervention areas chose “would call” again, and 12.8% (354/2767) of respondents changed their answer from “would call” to other answers, while 7.0% (193/2767) of respondents chose other answers again, and 11.3% (312/2767) changed their answer from other answers to “call an ambulance” (Figure 2).

### Association between knowledge of early stroke symptoms and intention to call an ambulance at stroke onset in the pre- and post-intervention surveys

The associations between knowledge of stroke symptoms and intention to call an ambulance in each survey are shown in Table 4 and Table 5. There was a difference in the association between knowledge of early stroke symptoms and intention to call an ambulance among the three surveyed areas in the pre-intervention survey (Table 4). In Akita, the intensive intervention area, four correct symptoms were associated with intention to call an ambulance, whereas this finding was not observed in the other areas. No decoy symptoms were associated with intention to call an ambulance in any areas in the pre-intervention surveys. In the post-intervention survey, although an increase in the number of correctly identified symptoms was significantly associated with intention to call an ambulance in all areas (from 4 correct symptoms to 5 correct symptoms in the intensive intervention area, and from 0 or 1 correct symptom to 3 correct symptoms in the other areas), choice of the decoy symptom “numbness of bilateral fingers” was also significantly associated with intention to call an ambulance in the moderate intervention area and in the control area (Table 5).

**Table 4.** Knowledge of stroke symptoms in those who would call an ambulance at stroke onset in the pre-intervention survey

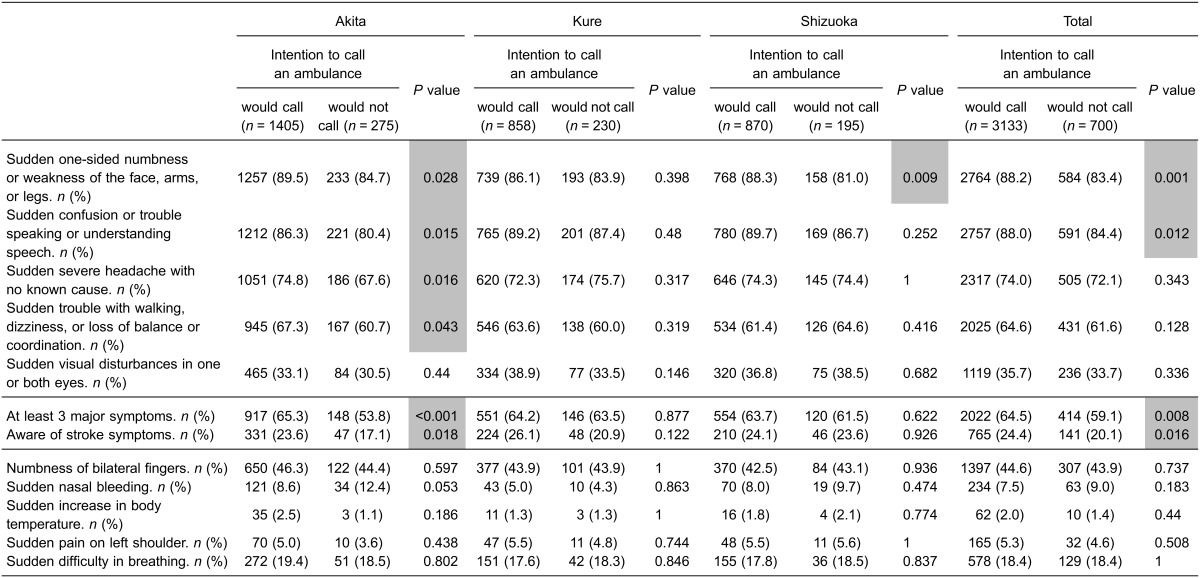

“At least three major symptoms”; those who selected at least three symptoms including “Sudden one-sided numbness or weakness of the face, arms, or legs”, “Sudden confusion or trouble speaking or understanding speech” and “Sudden severe headache with no known cause”. “Aware of stroke symptoms”; those who selected five correct symptoms. The data were analyzed using Fisher’s exact test with 2 tails, and are shown as numbers (%). Shaded columns show significant *P* value (<0.05).

**Table 5.** Knowledge of stroke symptoms in those who would call an ambulance at stroke in the post-intervention survey

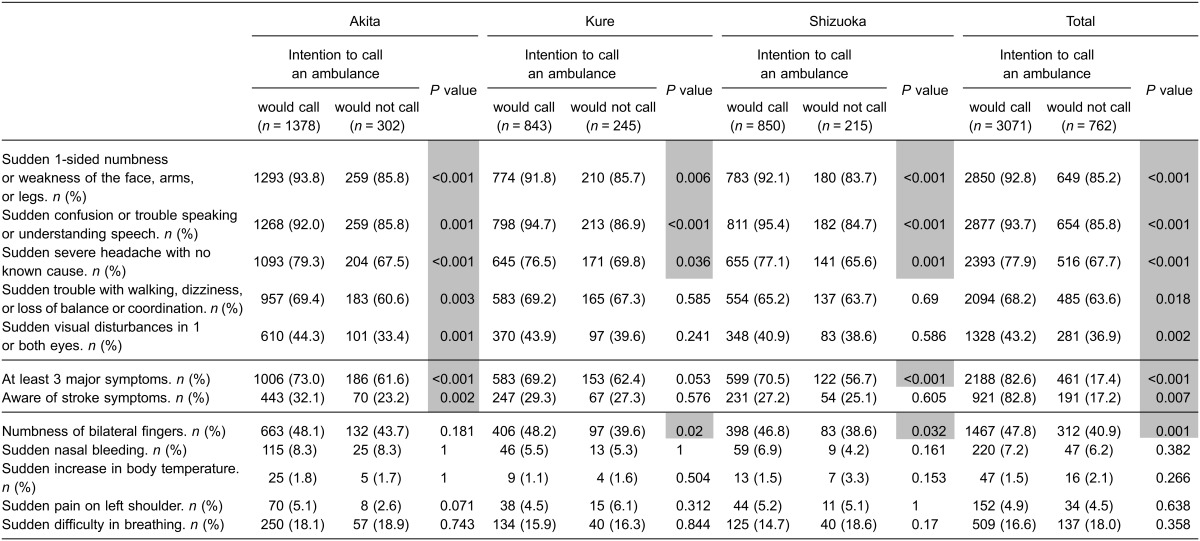

“at least three major symptoms”; those who selected at least 3 symptoms including “sudden one-sided numbness or weakness of the face, arms, or legs”, “sudden confusion or trouble speaking or understanding speech” and “sudden severe headache with no known cause”. “aware of stroke symptoms”; those who selected 5 correct symptoms. The data were analyzed using the Fisher’s exact test with 2 tails, and are shown as number (%). Shaded columns show significant *P* value (<0.05).

Furthermore, when the participants who had chosen ≤4 correct early stroke symptoms in the pre-intervention survey were selected and analyzed (1302 in Akita, 816 in Kure, and 809 in Shizuoka), a statistically significant association between choosing 5 correct early stroke symptoms in the post-intervention survey and the intention to call an ambulance was observed in Akita but not in the other two areas (*P* = 0.009 in Akita, *P* = 0.914 in Kure, and *P* = 0.823 in Shizuoka, using the two-tailed Fisher’s exact test).

## DISCUSSION

The present study, the first large-scale study to survey the same 3833 individuals before and after a 2-year educational campaign, demonstrated that intensive intervention could strengthen the association between knowledge of early stroke symptoms and intention to call an ambulance; that is, the campaign appears to have increased the proportion of participants who know the correct early stroke symptoms and understand the necessity of calling an ambulance in cases of suspected stroke. The intensive intervention also strengthened the association between knowledge of early stroke symptoms and intention to call an ambulance in the participants who had chosen ≤4 correct symptoms in the pre-intervention survey. In contrast, the present study did not show any increase in the proportion of those with an intention to call an ambulance after the intervention.

The findings about knowledge of early stroke symptoms in the pre-intervention survey were compatible with those from a previous study using a different sample from the same database^18^ (Table 1); however, there were some differences in knowledge of each symptom among participants in the three areas. In the previous study, there were significant differences in knowledge of “sudden confusion or trouble speaking or understanding speech”, “sudden one-sided numbness or weakness of the face, arms, or legs”, and “sudden trouble with walking, dizziness, or loss of balance or coordination” among participants in the three areas; in contrast, the present pre-intervention survey found significant differences in “sudden confusion or trouble speaking or understanding speech” and “sudden visual disturbances in one or both eyes”. This discrepancy is thought to be due to the sampling difference. In the previous study, participants who chose 5 correct answers at the pre-intervention survey were excluded because that analysis aimed to investigate the increase in the number of participants choosing correct early stroke symptoms after public education.

Whether educational campaigns can improve intention to call an ambulance at stroke onset remains controversial. Inconsistent findings may be explained by differences in the baseline proportions of respondents with intention to call an ambulance, which were high in the present study (78.9% to 83.6%). Indeed, similar findings to our own were observed in previous studies in which the baseline proportions of the respondents who would call an ambulance at stroke onset were also relatively high (74%^13^ and 81%^14^). In contrast, in many previous studies demonstrating an increase in the proportion of respondents who would call an ambulance at stroke onset, the baseline proportion was relatively low: 22.7% to 54.3% for symptoms for themselves^13^^,^^16^ and 29.0% to 74.0% for symptoms for another person.^15^^,^^16^ Considering these findings, the present results were considered to reflect a ceiling effect, although it is possible that the educational campaign, which only involved distribution of leaflets and booklets and holding lectures, might have been insufficient to increase the percentage of those who would call an ambulance at stroke onset.

Calling an ambulance does not always correspond to correct knowledge of early stroke symptoms. One of the reasons why the proportion of those who would call an ambulance at stroke onset was relatively high in the present study might be attributed to the emergency service system in Japan, which is so freely accessible that some people’s behavior has become a problem (ie, using an ambulance like a free taxi). In fact, there is a counter campaign announcing that “an ambulance is not a taxi,” because this kind of inappropriate use of the emergency system is exhausting emergency service workers. On the other hand, calling an ambulance is such an embarrassing thing for many people that they tend to hesitate to call an ambulance if they do not consider the symptoms to be serious enough. Therefore, in order to maintain appropriate use of emergency services, it is important to educate people to call an ambulance without delay based on the correct recognition of early stroke symptoms, including minor symptoms, and to differentiate non-early stroke symptoms. In addition, educational campaigns should be continuously performed because public awareness tends to decline over time in the absence of advertising.^21^^,^^22^

There are a number of limitations in this study. First, the baseline difference in the association between the knowledge of early stroke symptoms and intention to call an ambulance among participants in the three areas might have affected the results. In Akita, the intensive intervention area, a large proportion of the respondents with the intention to call an ambulance at stroke onset selected 4 correct early stroke symptoms, even in the pre-intervention survey. This is probably due to characteristics of Akita Prefecture itself, which has one of the highest stroke mortalities has in Japan (ie, a high percentage of respondents lived close to patients with a history of stroke; see Table 1) and where various public education campaigns on stroke had been performed prior to the present study. Although selecting similar areas might be desirable for this kind of study, it was difficult to select such areas because contamination may occur if the areas are too close to one another. Second, changes in the association between knowledge of early stroke symptoms and intention to call an ambulance were observed even in the control area. This background change was thought to be attributed to the influence of the stroke campaign conducted by AC Japan in every area, which is consistent with the findings in the previous study^18^ that the AC Japan campaign improved stroke knowledge. The improvement in knowledge of stroke symptoms was thought to be greater in the intervention areas than in the control area (Table 5); therefore, the effect of the stroke campaign conducted by AC Japan might have enhanced the effect of intervention in the intensive intervention area. Finally, in the present study, the direct association between knowledge of each stroke symptom and the intention to call an ambulance was not assessed. In order to evaluate such associations, specific questions assessing knowledge of each symptom should be asked (eg, “would you call an ambulance if you experienced a sudden visual disturbance in one or both eyes?”), and the proportion of participants responding correctly after the intervention should be analyzed. However, including these questions in our questionnaires was difficult because of the study design.

### Conclusions

Our findings suggest that intensive interventions are useful for strengthening the association between correct knowledge of early stroke symptoms and intention to call an ambulance, without strengthening the association between incorrect knowledge and intention to call an ambulance.
